# Dilated Thin-Walled Blood and Lymphatic Vessels in Human Endometrium: A Potential Role for VEGF-D in Progestin-Induced Break-Through Bleeding

**DOI:** 10.1371/journal.pone.0030916

**Published:** 2012-02-17

**Authors:** Jacqueline F. Donoghue, C. Jay McGavigan, Fiona L. Lederman, Leonie M. Cann, Lulu Fu, Eva Dimitriadis, Jane E. Girling, Peter A. W. Rogers

**Affiliations:** 1 Centre for Women's Health Research, Department of Obstetrics and Gynaecology and Monash Institute for Medical Research, Monash University, Clayton, Victoria, Australia; 2 Gynaecology Research Centre, Department of Obstetrics and Gynaecology, The University of Melbourne, Royal Women's Hospital, Melbourne, Victoria, Australia; 3 Embryo Implantation Laboratory, Prince Henry's Institute for Medical Research, Monash Medical Centre, Clayton, Victoria, Australia; University of Frankfurt - University Hospital Frankfurt, Germany

## Abstract

Progestins provide safe, effective and cheap options for contraception as well as the treatment of a variety of gynaecological disorders. Episodes of irregular endometrial bleeding or breakthrough bleeding (BTB) are a major unwanted side effect of progestin treatment, such that BTB is the leading cause for discontinued use of an otherwise effective and popular medication. The cellular mechanisms leading to BTB are poorly understood. In this study, we make the novel finding that the large, dilated, thin walled vessels characteristic of human progestin-treated endometrium include both blood and lymphatic vessels. Increased blood and lymphatic vessel diameter are features of VEGF-D action in other tissues and we show by immunolocalisation and Western blotting that stromal cell decidualisation results in a significant increase in VEGF-D protein production, particularly of the proteolytically processed 21 kD form. Using a NOD/scid mouse model with xenografted human endometrium we were able to show that progestin treatment causes decidualisation, VEGF-D production and endometrial vessel dilation. Our results lead to a novel hypothesis to explain BTB, with stromal cell decidualisation rather than progestin treatment per se being the proposed causative event, and VEGF-D being the proposed effector agent.

## Introduction

Progestins provide safe, effective and cheap options for contraception as well as the treatment of a variety of gynaecological disorders [1]. Although originally developed as a contraceptive, the levonorgestrel-releasing intra-uterine system (LNG-IUS) is also highly effective at controlling excessive menstrual blood loss in the majority of women treated [2]. Progestins are also used clinically in a number of other situations such as to provide relief from the symptoms of endometriosis by suppressing growth of the ectopic endometrium [3]. However, episodes of irregular endometrial bleeding or breakthrough bleeding (BTB) are a major unwanted side effect of progestin treatment, such that BTB is the leading cause for discontinued use of an otherwise effective and popular medication [4], [5]. Endometrial breakthrough bleeding is defined as any irregular or unpredictable bleeding that is not part of the normal menstrual process. Unlike menstrual bleeding, which occurs primarily from the spiral arterioles in response to falling levels of estrogen and progesterone [6], BTB occurs from the endometrial capillaries and smaller vessels [7].

Understanding of the cellular mechanisms underlying BTB remains elusive because of both the range of hormonal conditions under which it can occur and the high degree of variability between women in terms of their endometrial response to exogenous hormones and their susceptibility to BTB. Thus, while administration of exogenous progestins will increase the incidence of BTB, the link between hormones and BTB is not direct. Numerous studies over the past 20 years have been undertaken with the aim of developing a better understanding of the local endometrial mechanisms responsible for BTB. Some of factors that have been proposed as playing a role in BTB include endometrial epithelial integrity [8], altered MMP levels [9], altered leucocyte populations [10], reduced vascular pericyte coverage [11], [12], and increased vascular fragility [13]. Despite this work, widespread consensus as to mechanisms that might be responsible for BTB does not exist yet within the field.

Decidualisation is a hormonally regulated cellular differentiation process that occurs in the endometrial stroma of most mammals prior to and during placentation. It is accompanied by significant vascular remodelling of both blood and lymphatic vessels [14], [15] as part of the process of ensuring an adequate blood supply to the placenta. In humans, decidualisation commences during the mid-late secretory phase under the influence of luteal progesterone regardless of whether embryo implantation has occurred. However, if implantation does not occur, circulating progesterone levels drop rapidly and the decidualised tissue is shed a few days later during menstruation.

Endometrium exposed to progestins undergoes a well characterised series of morphological changes that includes stromal cell pseudo-decidualisation, epithelial cell regression [16] and the appearance of abnormally dilated and thin-walled endometrial vessels [7], [17], [18], [19], [20], [21], [22]. While it has been proposed that the abnormally dilated, thin walled vessels found in progestin-exposed endometrium may contribute to BTB through increased vascular fragility [13], there is currently a lack of information characterizing these vessels, and little insight as to the mechanisms behind their formation. If these dilated, thin-walled vessels do play a role in BTB, a better understanding of how they are formed may assist in identifying new avenues for treating this troublesome side-effect of progestin treatment.

The overall aim of this study was to investigate potential mechanisms by which progestin administration results in the formation of abnormally dilated and thin-walled endometrial vessels.

We were able to demonstrate that a significant proportion of the dilated, thin walled vessels in progestin treated human endometrium are lymphatics and not blood vessels. Both lymphatic and blood vessels show marked increases in diameter following progestin treatment, while lymphatics in the functional layer are the only vessels to show a moderate increase in number. Increased blood and lymphatic vessel diameter, but not number, are features of VEGF-D action in other tissues [23] and we show by immunolocalisation and Western blotting that stromal cell decidualisation results in a significant increase in VEGF-D protein production, particularly of the biologically active 21 kD form. We then use a NOD/scid mouse model with xenografted human endometrium to show that progestin treatment causes decidualisation, VEGF-D production and endometrial vessel dilation. Our results lead to a novel hypothesis to explain BTB, with stromal cell decidualisation rather than progestin treatment per se being the proposed causative event, and VEGF-D being the proposed effector agent.

## Results

### Endometrial Blood and Lymphatic Vessels have Increased Diameters in Women Exposed to Intra-Uterine Levonorgestrel

**Figure 1:**
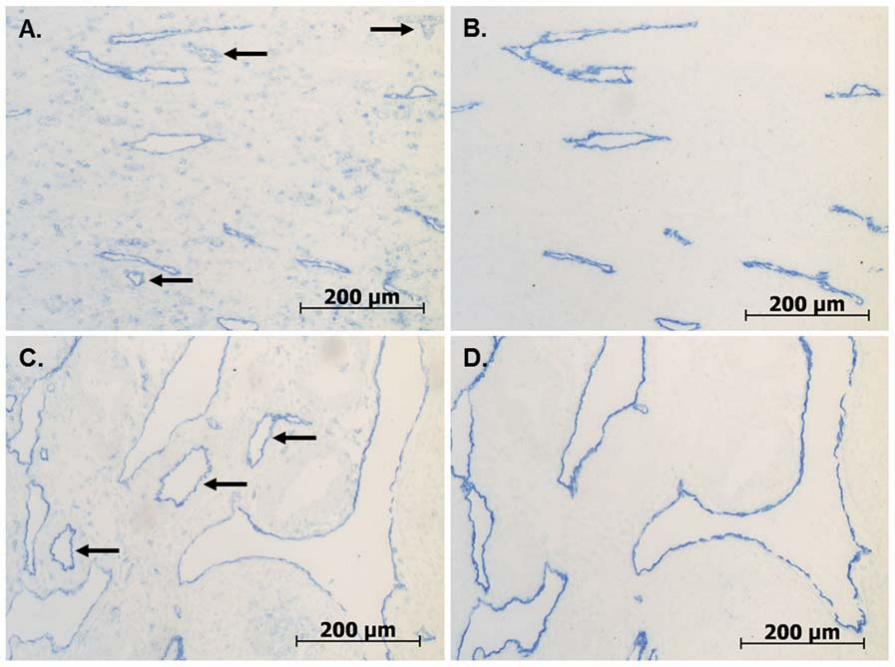
Progestin-induced endometrial blood and lymph vessel dilatation. Enlarged blood and lymphatic vessels are present in the endometrium of women treated with LNG-IUS prior to hysterectomy for heavy menstrual bleeding. (*A–B*) Untreated control samples. (*C–D*) LNG-IUS treated samples. Sections have been immunostained with CD31 (*A, C*), which labels both blood and lymphatic endothelial cells, or D2-40 (*B, D*), which labels lymphatic endothelial cells only. Black arrows: blood vessels.

Full-thickness endometrial sections from women with heavy menstrual bleeding who had either been treated with a levonorgestrel intrauterine system (LNG-IUS) (N = 16) or who were untreated controls (N = 16) [22], were immunostained with antibodies to CD31 (labels both lymphatic and blood endothelial cells) or D2-40 (labels lymphatic endothelial cells only). Blood vessel counts were made from CD31 immunostained sections by subtracting the D2-40 immunostained vessel count made on a serial section. Endometrial blood and lymphatic vessels were readily identifiable in all sections following immunostaining (Fig. 1). Most of the microvessels were of normal appearance in both the LNG-IUS treated tissue and the controls. However, in the levonorgestrel treated tissue, but not the control tissue, large, thin walled vessels were also observed. Immunohistochemistry with D2-40 revealed that some of these vessels were lymphatics. In LNG-IUS treated tissue, the large or dilated lymphatic and blood vessels appeared to be mostly located in the functional layer, although a few were also seen in the basal layer.

**Figure 2:**
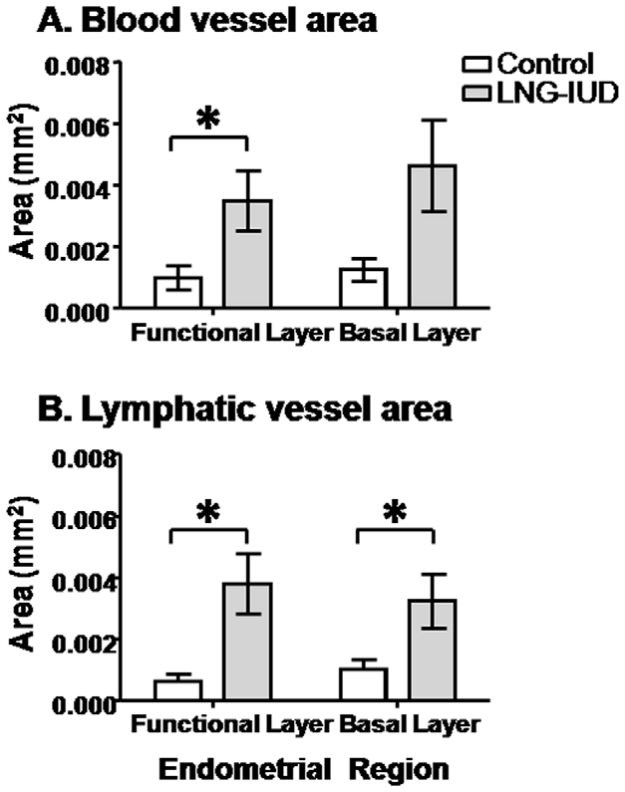
Increased blood and lymphatic vessel area after progestin exposure. Blood and lymphatic vessel cross sectional areas are increased in endometrium from women treated with LNG-IUS prior to hysterectomy for heavy menstrual bleeding. (A) The mean area of the five largest blood vessels in the functional layer and the basal layer in untreated control (white bars) and LNG-IUS treated (grey bars) endometrium. (B) The mean area of the five largest lymphatic vessels in the functional layer and the basal layer of untreated control (white bars) and LNG-IUS treated (grey bars) endometrium. Columns represent means ± SE. *, *P*<0.05.

Using 2-way ANOVA, the vessel cross sectional area of the 5 largest blood vessels was significantly greater in endometrium from women treated with LNG-IUS compared to controls (F_(1,59)_ = 12.693, *P* = 0.001) (Fig. 2*A*). Tukey's posthoc tests showed that this observation was true in the functional layer (Control: 1.0×10^−3^±3.8×10^−4^ mm^2^; LNG-IUS: 3.5×10^−3^±9.7×10^−4^ mm^2^; *P* = 0.026) but not the basal layer (Control: 1.2×10^−3^±3.7×10^−4^ mm^2^; LNG-IUS: 4.6×10^−3^±1.4×10^−3^ mm^2^; *P* = 0.165). A similar analysis of the five largest lymphatic vessels showed a significant increase in area for LNG-IUS versus control treated tissues (F_(1,60)_ = 19.569, *P* = 0.001) (Fig. 2*B*). This was the case in both the functional layer (Control: 6.4×10^−4^±2.3×10^−4^ mm^2^; LNG-IUS: 3.8×10^−3^±9.8×10^−4^ mm^2^; *P* = 0.01) and also the basal layer (Control: 1.0×10^−3^±3.1×10^−4^ mm^2^; LNG-IUS: 3.2×10^−3^±8.8×10^−4^ mm^2^ *P* = 0.021).

**Figure 3:**
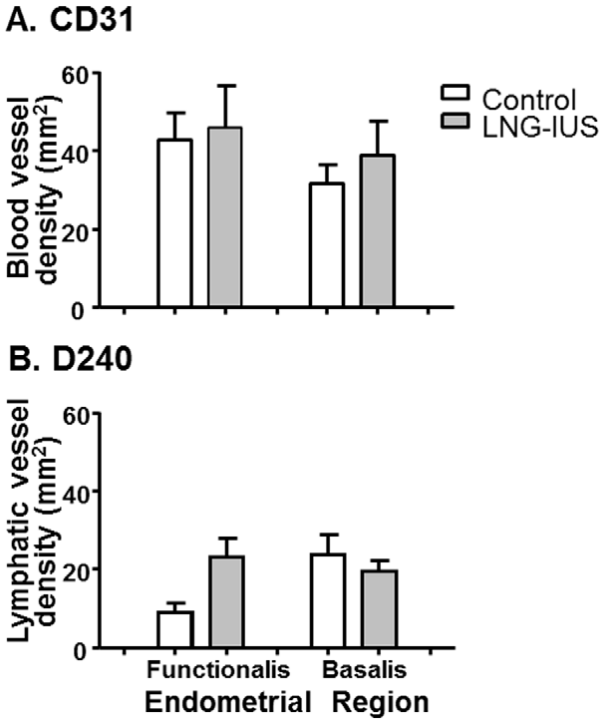
Endometrial blood and lymphatic vessel density after progestin exposure. Blood and lymphatic vessel densities do not change in endometrium from women treated with LNG-IUS prior to hysterectomy for heavy menstrual bleeding. (A) Vascular density of blood vessels in untreated control (white bars) and LNG-IUS treated (grey bars) samples of the functional layer and the basal layer. (B) Lymphatic vessel density in untreated and LNG-IUS treated endometrial samples. Columns represent means ± SE. *, *P*<0.05.

In contrast to vessel cross sectional areas, there was no difference in endometrial blood vessel density (F_(1,53)_ = 1.08, *P* = 0.304 or lymph vessel density (F_(1,63)_ = 2.384, *P* = 0.128) between control or LNG-IUS treated tissues (Fig. 3). Also, there was no overall significant difference in vessel density between the functional layer vs. the basal layer for blood vessels (Functional Layer: Control: 42±6.9 vessels/mm^2^; LNG-IUS: 45±10.7 vessels/mm^2^ Basal Layer: Control: 31±4.8 vessels/mm^2^; LNG-IUS: 39±8.6 vessels/mm^2^; F_(1,53)_ = 0.342, *P* = 0.562) or lymphatics (Functional Layer: Control: 9±2.5 vessels/mm^2^; LNG-IUS: 23±4.8 vessels/mm^2^ Basal Layer: Control: 24±5.0 vessels/mm^2^; LNG-IUS: 20±2.6 vessels/mm^2^; F_(1,63)_ = 2.779, *P* = 0.101).

### Human Endometrial Stromal Cells Show Increased Expression of VEGF-D Following Decidualisation

**Figure 4:**
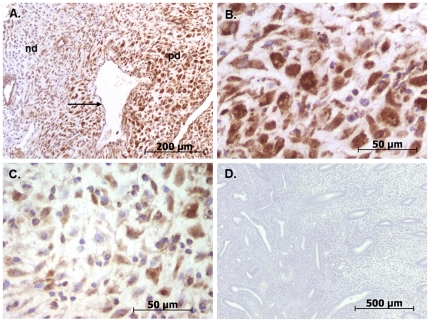
VEGF-D immunostaining in endometrial decidual cells. Distinct VEGF-D immunostaining (brown) is present in pre-decidual cells of endometrium from women treated with LNG-IUS prior to hysterectomy. (A) Micrograph illustrating difference in VEGF-D immunostaining (brown) intensity in stromal pre-decidualised cells (pd) compared to non-decidualised cells (nd). (B) Higher power image of pre-decidualised stromal cells showing VEGF-D immunostaining in brown. (C) Higher power image of non-decidualised stromal cells showing reduced VEGF-D immunostaining. (D) Isotype matched negative control. Black arrow: blood vessel. (sections are lightly counterstained with haematoxylin to identify cell nuclei in blue).

Increased blood and lymphatic vessel diameters are a recognised consequence of elevated tissue expression of VEGF-D [23]. Immunohistochemical and Western blotting techniques were used to investigate whether VEGF-D protein levels are increased in decidualised endometrial stromal cells. Focal areas of pre-decidualisation were common in endometrium treated with LNG-IUS (N = 16) but not in endometrium from controls (N = 16). Pre-decidual cells showed intense immunostaining for VEGF-D (Fig. 4) but not VEGF-C (data not shown). There was a clear increase in VEGF-D immunostaining intensity in pre-decidual cells compared to non-decidualised stromal cells in areas where these two cell types appeared together (Fig. 4*A* and 4*C*).

**Figure 5:**
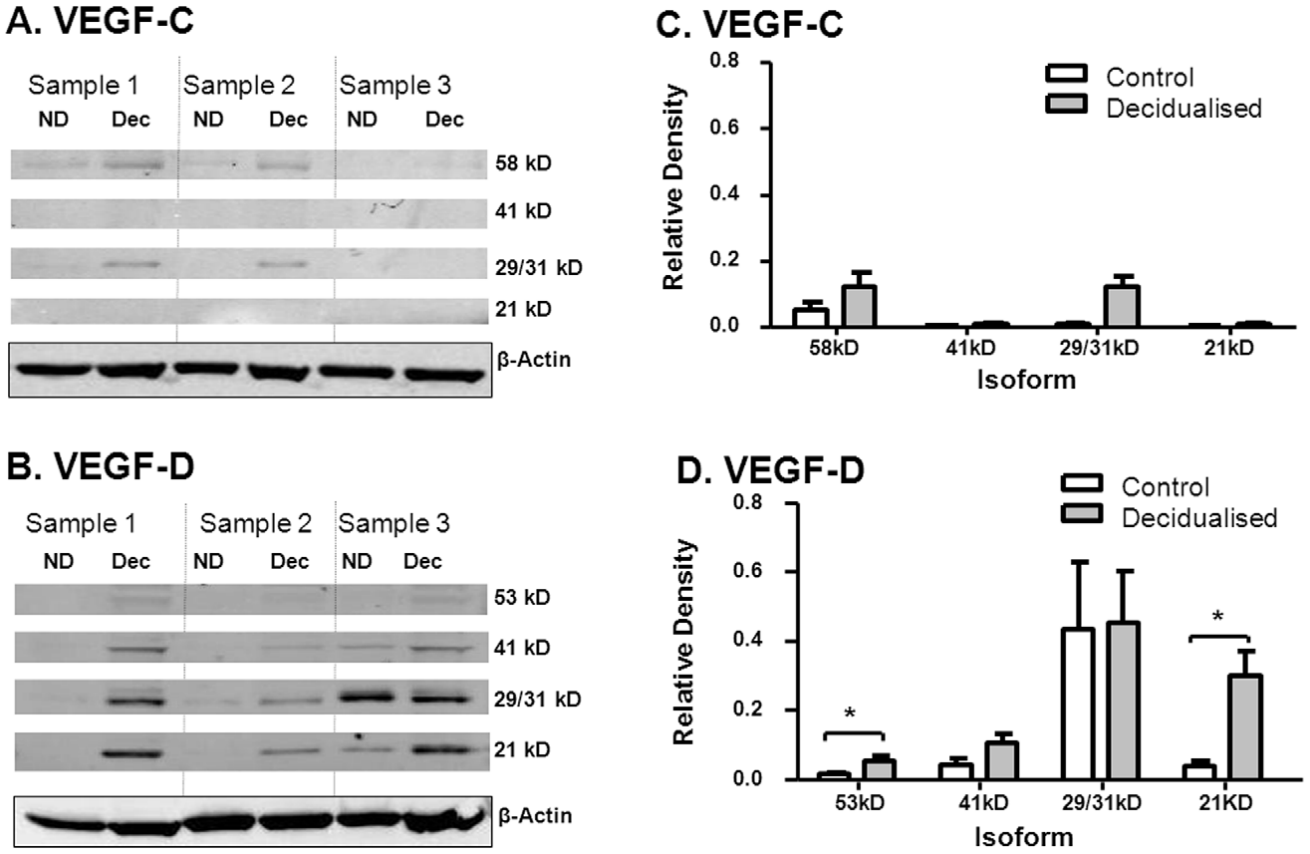
VEGF-D protein in endometrial decidual cells. Western analysis of vascular endothelial cell growth factor-C (VEGF-C) and VEGF-D peptide expression in primary cultures of human endometrial stromal cells (HESC). Panels A and B show representative data from 3 samples. Panels C and D show combined densitometry data from all 5 samples. (A) VEGF-C peptide expression (58, 41, 29/31 and 21 kD) in HESC (ND) and decidualised HESC (Dec). (B) VEGF-D peptide expression (53, 41, 29/31 and 21 kD) in HESC and decidualised HESC. (C–D) Densitometry of VEGF-C and VEGF-D peptide expression. White bars representing control non-decidualised HESC and black bars representing decidualised HESC. Columns represent means ± SE; * *P*<0.05.

To further explore whether the process of decidualisation results in elevated expression of VEGF-C and VEGF-D protein, Western analysis was performed on human endometrial stromal cells before and after decidualisation in vitro (N = 5 samples). Human endometrial stromal cells showed evidence by Western analysis for increased expression of both VEGF-C and VEGF-D after decidualisation in vitro (Fig. 5*A*, 5*B* and Fig. S1). Up to seven VEGF-D polypeptides were detected including 53, 44, 41, 29/31 and 21 kD [24]. There was a significant increase in VEGF-D expression in decidualised versus non-decidualised cells (F_(1,16)_ = 22.2, *P* = 0.001) with a significant variation among different isoforms (F_(3,16)_ = 4.38, *P* = 0.02). Paired T-tests were significant after Bonferoni correction for the 53 kD (3.5-fold increase in relative density, *P* = 0.002) and 21 kD (8.2-fold increase in relative density, *P* = 0.005) isoforms, but not for the 41 (*P* = 0.05, which fails to reach *P* = 0.0125 as required by the Bonferoni correction) and 29/31 kD (*P* = 0.26) isoforms. The increase in the 21 kD isoform is of particular relevance as following proteolytic processing this is the most biologically active isoform with the highest affinity for VEGF receptor-3 (VEGFR-3), which in the adult is found on lymphatic endothelial cells [25]. VEGF-C protein was detected as 58, 43, 29/31 kD polypeptides [26]. Densitometry showed there was a significant increase in VEGF-C expression in decidualised versus non-decidualised cells by 2-way mixed ANOVA (F_(1,16)_ = 25.7, *P* = 0.001), as well as a significant variation among different isoforms (F_(3,16)_ = 4.93, *P* = 0.013). Paired T-tests for individual isoforms failed to reach significance after Bonferoni correction (Note: to reach significance after the Bonferoni correction, a P-value of 0.0125 is required: 58 KD: *P* = 0.024, 43 kD: *P* = 0.21, 29/31 kD: *P* = 0.023; 21 kD: P = 0.50; Fig. 5*C*).

### Exogenous Progestin Produces a Pre-decidual Response in Human Endometrial Xenografts Resulting in VEGF-D Expression and Increased Lymphatic Vessel Diameters

**Figure 6:**
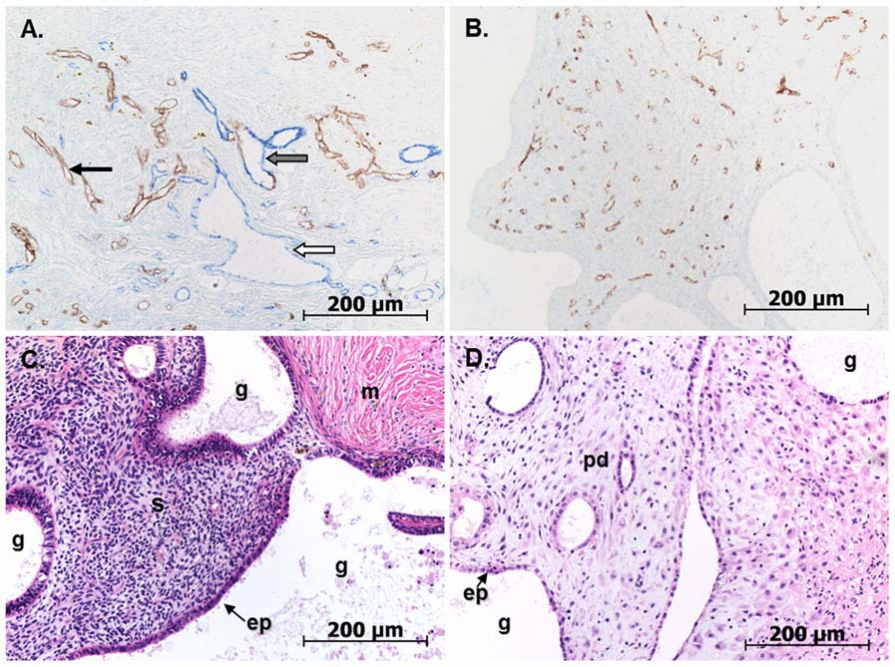
Human endometrial xenografts in NOD/scid mice. Representative micrographs of human endometrial xenografts taken from NOD/scid mice after 6 weeks growth at a subcutaneous site. (A) A mixture of human and mouse vessels at the edge (capsule) of the xenograft shown by double immunostaining with anti-human CD31 (brown, black arrow) and anti-mouse CD31 (blue, white arrow). Note the vessel of mixed human and mouse origin (grey arrow). (B) The central portion of the xenograft populated almost exclusively with vessels of human origin (double immunostaining with anti-human CD31 in brown and anti-mouse CD31 in blue). (C) Routine haematoxylin and eosin stained xenografts treated with estradiol valerate developed densely packed fibroblast-like stromal cells and cuboidal to columnar epithelial cells. (D) Routine haematoxylin and eosin stained xenografts grafts treated with MPA displayed pre-decidualised stromal cells with flattened epithelial cells. ep: epithelium, g: glands, m: myometrium, pd: pre-decidual stroma, s: stroma.

**Figure 7:**
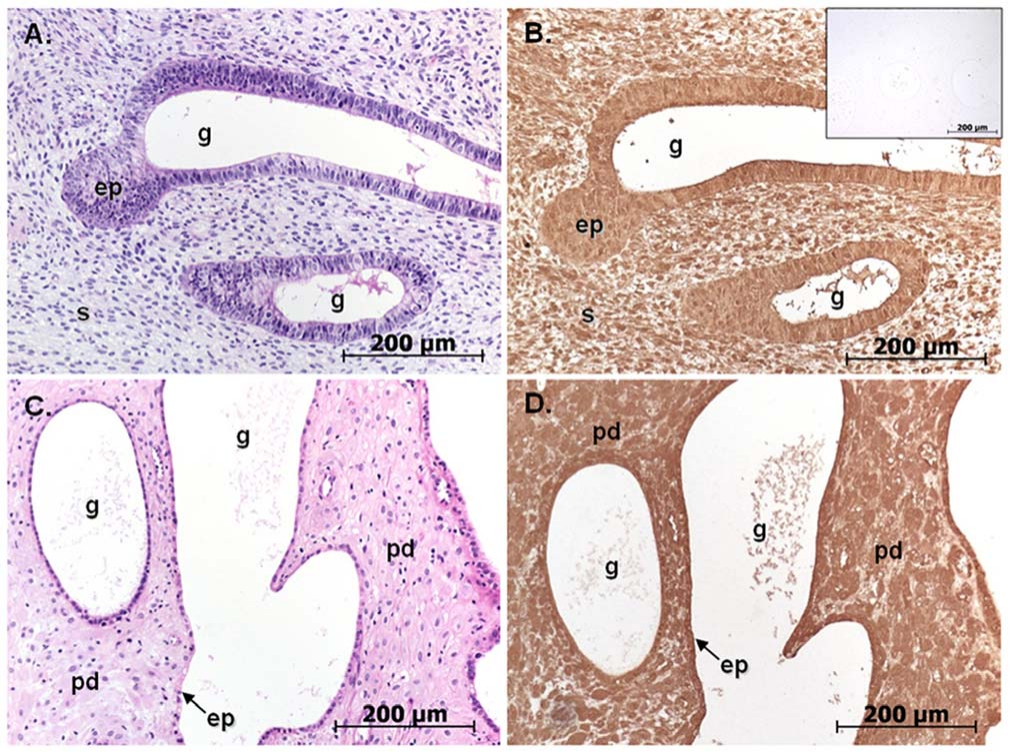
VEGF-D immunostaining of xenograft decidual cells. Representative micrographs of human endometrial xenografts from NOD/scid mice treated with estradiol valerate (A,B) or medroxyprogesterone acetate (C,D). Serial sections were stained with haematoxylin and eosin (A,C) or immunostained with VEGF-D (B,D). Inset in (B): Isotype matched negative control. Note the pre-decidual cells in MPA-treated sections. ep: epithelium, g: glands, pd: pre-decidual stroma, s: stroma.

To further explore the role of exogenous progestin in causing lymphatic vessel dilatation through decidualisation-dependent expression of VEGF-D, a human endometrial xenograft model in NOD/scid mice was utilised. A total of n = 4 human samples were subdivided and grafted into NOD/scid mice that were subsequently treated with estradiol valerate only, or estradiol valerate and medroxyprogesterone acetate (MPA). The estrogen component of this treatment maximizes xenograft establishment, vascularisation and survival, and the progestin recreates the hormonal status that exists in women using progestin contraceptives.There was a 100% recovery of graft tissue at the end of the experimental period. There were no obvious macroscopic differences between the treatment groups with all grafts becoming encapsulated by extracellular matrix. The endometrial vasculature was composed of both blood and lymphatic vessels which were predominantly of human origin (92±3%) with a small number of murine vessels also infiltrating the endometrial tissue (8±3%) (Fig. 6*A* and 6*B*). All subsequent analysis of blood and lymphatic vessels only included vessels that exhibited positive immunostaining with human anti-CD31 antibody. The endometrial xenografts that received estrogen-only contained stromal cells that were densely packed and fibroblast-like in appearance while the glandular epithelium was cuboidal or columnar (Fig. 6*C* – H&E staining). In contrast, the progestin-treated xenografts exhibited pre-decidualised stromal cells with flattened glandular epithelial cells (Fig. 6*D* – H&E staining). To confirm that decidual cells in progestin treated xenografts expressed VEGF-D, immunostaining was performed. VEGF-D immunostaining was intense in decidualised tissue in xenografts (Fig. 7), and was also apparent in epithelial cells.

**Figure 8:**
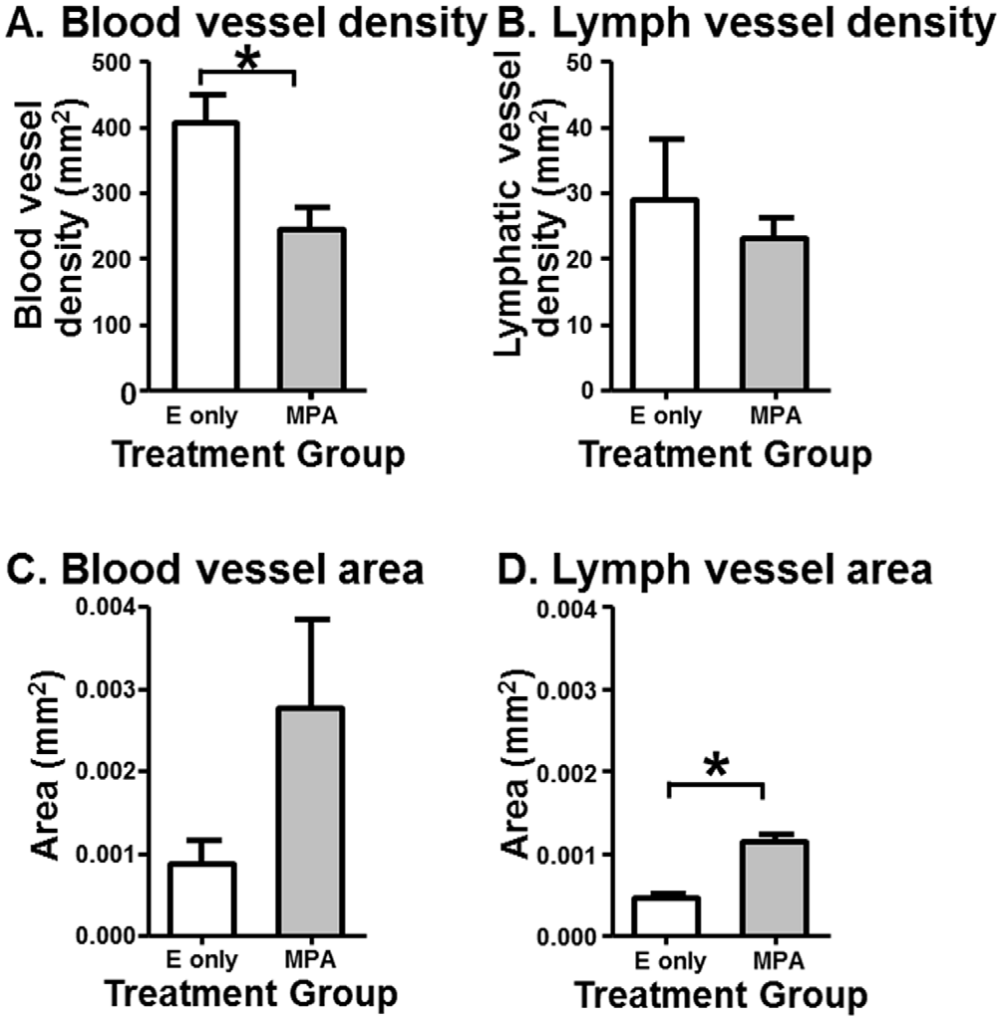
Progestin effects on xenograft vessel density and area. Blood vessel density decreases and lymphatic vessel cross sectional vessel area increases in human endometrial xenografts treated with medroxyprogesterone acetate (MPA). (A) Blood vessel density and (B) lymphatic vessel density of untreated (E only) and treated (MPA) xenografts. (C) Blood vessel and (D) lymphatic vessel cross sectional area of E only and MPA treated xenografts. Bars represent means ± SE. * *P*<0.05.

Blood and lymphatic vessel density and cross sectional area were evaluated in progestin-treated (n = 4) and control xenografts (n = 4) following immunostaining with antibodies to CD31 and D2-40. There was a significant increase in cross-sectional area of the 5 largest lymph vessels in the progestin treated xenografts (4.6×10^−4^±5.0×10^−5^ vessels/mm^2^) when compared to the estrogen-only treated xenografts (1.1×10^−3^±1.0×10^−4^ vessels/mm^2^) t_(6)_ = −5.873, *P* = 0.001) (Fig. 8*D*). Xenografts treated with progestin for 4 weeks (245±35.9 vessels/mm^2^) had significantly reduced blood vessel density when compared to xenografts that received estrogen-only (406±44.3 vessels/mm^2^) for the same length of time (t_(6)_ = 2.848, *P* = 0.029) (Fig. 8*A*). There was no difference in lymph vessel density between treatment groups (estrogen-only: 29±9.2 vessels/mm^2^; progestin: 23±3.1 vessels/mm^2^; t_(6)_ = 0.619, *P* = 0.559) (Fig. 8*B*). There was also no significant difference in the cross-sectional area of the 5 largest blood vessels between the treatment groups (estrogen-only: 8.7×10^−4^±3.0×10^−4^ vessels/mm^2^; progestin: 2.8×10^−3^±1.1×10^−3^ vessels/mm^2^; t_(6)_ = −1.681, *P* = 0.179) (Fig. 8*C*).

## Discussion

In this study, we make the novel finding that the large, dilated vessels characteristic of progestin-treated endometrium include both blood and lymphatic vessels (Fig. 1). That both blood and lymphatic vessels exhibit abnormal morphology and dilatation in response to progestin is consistent with a regulatory mechanism common to both vessels types. Evidence for such a mechanism comes from studies evaluating the therapeutic potential of human VEGF family members using adenoviral gene transfer in rabbit hind limb skeletal muscle [23]. The VEGF family of angiogenic and lymphangiogenic peptides includes VEGF-A, VEGF-B, VEGF-C, VEGF-D and placental growth factor [27]. VEGF-C and VEGF-D act by binding and activating the tyrosine kinase receptors VEGF receptor-2 (VEGFR-2) and VEGFR-3, which in the adult are found predominantly on blood and lymphatic endothelial cells, respectively [28], [29]. VEGF-C and VEGF-D differ from other members of the mammalian VEGF family due to the presence of pro-peptides at both the N- and C-termini of the conserved VEGF homology domain [26]. Cleavage of the pro-peptides by proteases such as plasmin modulates bioactivity of both molecules, increasing affinity for both VEGFR-2 and VEGFR-3 [25], [26], [30]. In the rabbit hind limb skeletal muscle study, both VEGF-A and the proteolytically processed 21 kD form of VEGF-D caused a remarkable enlargement of microvessels, however, while VEGF-A only affected blood vessels, VEGF-D enlarged both blood and lymph vessels [23]. VEGF-A also moderately increased capillary density, whereas VEGF-D induced angiogenesis was more diffuse. VEGF-D has also been shown to induce vessel enlargement in mouse hind limb [23], [31], rat cremaster muscle [32], pig heart [33], mouse skin [34] and mouse uterine horn [35]. The remarkable similarity in the changes to the blood and lymph microvessels in our study and those previously published provided the impetus to further investigate a role for VEGF-D in the progestin treated human endometrium.

Progestin-treated endometrium usually exhibits areas of decidualisation, especially in the first few months of exposure before endometrial regression has occurred [17], [18], [36]. However, in contrast to the normal menstrual cycle, these decidual cells are maintained by the exogenous progestin for a relatively long period of time in the absence of implantation or placentation. Given that decidual cells play a central role in placentation, it is reasonable to speculate that the pathological vascular changes seen in progestin-affected endometrium are the consequence of normal vascular remodelling for placentation that has become defective through a lack of implantation. The observation that BTB occurs with nearly all types of progestin is consistent with the hypothesis that it is the prolonged presence of decidual cells that is causing BTB, rather than a direct effect of the progestins on blood vessels. In a mouse model of BTB it has been shown that endometrial breakdown following prolonged progestin treatment is dependent on decidualisation [37]. Although this study does not link decidualisation directly to a mechanism that causes vascular dilatation, it points towards a relationship between decidualisation and BTB.

While VEGF-D is a highly plausible candidate for causing the microvascular changes seen in progestin-exposed endometrium, decidual cells have upregulated expression of many other proteins, including connexin 43, prolactin, IL-6 and VEGF-A [38]. It is possible that one or several of these other agents may play contributory roles in causing BTB. We have previously investigated VEGF-A expression by immunohistochemistry in decidualised and non-decidualised endometrial stromal cells of women with BTB following progestin-only contraception and found no difference [39], despite the fact that it is up-regulated in in vitro decidualised stromal cells [38].

While the current study establishes a potential mechanistic link between progestins, decidualisation and endometrial microvessel dilatation, the link between dilated endometrial vessels and BTB remains more speculative. Further, if a relationship between dilated endometrial vessels and BTB does exist, we would assume that it is the blood vessels and not the lymphatic vessels that are the primary cause. There are several published studies that do support a link between vessel dilatation and BTB. Most significantly, dilated endometrial vessels, which are a common occurrence with systemic or intrauterine progestin exposure, have been shown to demonstrate increased fragility in hysteroscopic studies [13], [19], [40]. Additionally, it has been shown that perivascular α-smooth muscle actin (αSMA) is reduced around the endometrial vessels of progestin users with BTB compared to those with no bleeding problems [11]. Reduced αSMA indicates a loss of perivascular support cells, specifically pericytes and vascular smooth muscle cells, a process which could be expected to contribute significantly to increased vascular fragility and BTB. Other groups have also reported a reduction in endometrial vessel αSMA coverage following exposure to the progestin levonorgestrel [12]. This study suggested that time-dependent changes in blood vessel number, area, density and maturation following exposure to LNG-IUS may explain the early transient increase in BTB. In a further study examining the link between BTB and endometrial vessel structure, it was reported that in women with BTB after 12 months of progestin treatment, significantly greater numbers of enlarged, distended vessels were present in tissues taken from bleeding sites compared with non-bleeding sites [41]. These authors suggested that chronic over expression of endometrial Tissue Factor (TF) at the bleeding sites might be promoting aberrant angiogenesis, resulting in the enlarged and fragile vessels with increased risk of bleeding.

In the current study we used a xenograft model to investigate the progestin-related changes to human endometrium caused by medroxyprogesterone acetate treatment *in vivo*. Our results parallel a recent study that used LNG to treat human endometrial xenografts for 4 weeks in SCID mice [42]. In both studies, 4 weeks of progestin exposure caused heterogeneous decidualisation and an increase in vessel diameter in subcutaneous endometrial explants. Additionally, both models retained a greater than 90% presence of human vessels within the xenograft with minimal invasion of host vasculature [43]. The similar observations from these 2 independent studies confirm the utility of the xenograft model for studying human endometrial microvascular function.

The current study identifies both decidual cells and VEGF-D as potential targets for development of therapies for BTB. If decidual derived VEGF-D is the agent responsible for BTB, then a number of interventions might be possible. These include developing progestins that minimise or avoid decidualisation, developing agents that block progestin-induced decidualisation, and using inhibitors of VEGF-D to prevent endometrial microvascular dilatation.

Despite BTB having been a significant clinical problem for many years, advances in understanding the mechanisms that cause BTB have been limited. The lack of a readily available animal model that reliably replicates progestin-induced BTB is probably the single most critical issue responsible for this lack of progress. The absence of an accepted model means that mechanistic studies can only be performed in humans, which limits significantly the research that can be undertaken. The xenograft model used the current study does not exhibit BTB, despite replicating many of the changes seen in human endometrium exposed to progestins. Future progress in understanding BTB is most likely to come from a combination of indirect mechanistic studies supported by observational and correlational data from human subjects with BTB.

In conclusion, we have identified dilated lymphatics in progestin exposed human endometrium from women with BTB, which in conjunction with dilated blood vessels identifies VEGF-D as a potential causative agent. Further studies show that VEGF-D expression increases as endometrial stromal cells undergo decidualisation, and that this chain of events can be replicated in human endometrial xenografts in NOD/scid mice treated with progestin. Taken together these data implicate decidual cell-derived VEGF-D as a causative factor in progestin-induced BTB.

## Materials and Methods

### Human samples and specimen collection

Fixed and wax embedded human uterine tissue blocks from a previously published study [22] were obtained from women that had been exposed to LNG-IUS as treatment for heavy menstrual bleeding. Briefly, women who opted for a hysterectomy due to continued heavy menstrual bleeding following standard treatment options, were randomly allocated into LNG-IUS (*n* = 16, mean age 38.6 years) or control groups (*n* = 16, mean age 39.9 years). LNG-IUS were in place for up to 156 days prior to surgery. For the xenograft study, uterine biopsies were obtained following written informed consent (this work was approved by Southern Health Human Research and Ethics Committee under Project No. 08176B) from 4 patients aged 48.7±1 year undergoing hysterectomy for prolaspe or fibroids. Each sample was cut down to the endometrial myometrial boarder and divided into 8 grafts approximately 1×1×0.5 cm in size.

### Animals and Hormone Treatments

For xenograft studies, NOD/SCID female mice (*n* = 32; 6–8 weeks) received a single graft in a pocket created on the right side of the back flank (This work was carried out in accordance with the Australian Code of Practice for the Care and Use of Animals for Scientific Purposes (2004) and approved by Monash Medical Centre Animal Ethics Committee A, approval No. 2008/14). Following closure of the wound site, mice received 1 µg 17β-estradiol valerate in oil (subcutaneous) every fourth day until experiment completion. After two weeks of graft establishment, mice were divided into two groups and received a subcutaneous silastic tube implant [44]. The 17β-estradiol valerate group (Control) received an empty implant while the medroxyprogesterone acetate (MPA-Progestin) group received an implant containing 20 mg of MPA. Progestin treatment was continued for 4 weeks until graft harvest and fixation.

### Sample collection and Immunohistochemistry

Patient samples and xenografts were formalin fixed and paraffin embedded for immunohistological analysis. Tissue sections (5 µm) mounted on silane coated slides were dewaxed and rehydrated. Following antigen retrieval, endogenous peroxide block and protein block, immunolocalisation of human vascular endothelial cells (CD31: Clone JC 70A, Dako Cytomation, CA, USA,), human lymphatic endothelial cells (anti-Podoplanin: Clone D2-40, Signet Laboratories, MA, USA) [45], mouse vascular endothelial cells (CD31: #553370, BD Pharmingen, NJ, USA) and human VEGF-D (#AF469, R&D Systems MN USA) was performed as previously described [35]. Using a 20× objective lens, the five largest vessels within the endometrial component of the tissue were identified and measured in mm^2^ using the Analytical Imaging System (AIS) (Imaging Research Inc. GE Healthcare Bio-Sciences, NSW, Australia). We found this approach to be a simple and statistically robust morphometric method for objectively demonstrating the existence of the abnormally dilated and thin-walled vessels found in progestin-exposed endometrium.In xenograft samples, vessel density was determined by calculating the average number of vessels from 4 separate endometrial areas. Statistical analysis was performed using SPSS for Windows, Version 11.0.0 (SPSS Inc., Chicago, IL, USA). Vessel cross sectional area and vessel density of the functional layer and basal layer vessels in the endometrium of women +/− LNG-IUS was determined by 2-way ANOVA on logged data followed by Tukeys HSD post hoc analysis. Statistical analysis of xenograft +/− MPA vessel density and diameter was determined by Student t test. Values are presented as mean ± SE unless otherwise stated. A p value <0.05 was considered significant.

### Western Blotting

Primary isolated human endometrial stromal cells (from n = 5 endometrial biopsies) were decidualised following incubation with estradiol 17β (10^−8^ mol/l) and progesterone (10^−7^ mol/l) as previously described [46]. Protein from cell lysates (30 µg) were resolved on a 4–12% NuPAGE gel (Invitrogen Corporation; CA, USA). Following transfer to PVDF membranes, proteins were probed for VEGF-C (1∶200; AF286 R&D Systems; MN, USA), VEGF-D (1∶200; AF752 R&D Systems) or β-actin (1∶4000; Sigma Aldrich; NSW, Australia) overnight at 4°C. Signal was enhanced with fluorescent secondary antibodies (1∶20,000; AlexaFluor 680 Invitrogen or IRD 800 LI-COR Biosciences; NE, USA). Membranes were scanned and densitometry determined by the Odyssey® Infrared Imaging System (LI-COR Biosciences). The relative densitometry for each peptide band was normalised to the β-actin and expressed as a percentage of β-actin. Densitometry data from VEGF-C and VEGF-D western blots were analysed using a 2-way Mixed ANOVA followed by posthoc analysis using paired T-tests with Bonferoni corrections to correct for multiple comparisons.

## Supporting Information

Figure S1 Representative western blots of (A) VEGF-C and (B) VEGF-D protein expression in primary cultures of decidualised (Dec) and non-decidualised (ND) human endometrial stromal cells. White arrows indicate the reported isoforms including the C-VHD-N full length receptor (VEGF-C: 58 kD; VEGF-D: 53 kD; dimers at 105 kD), the VHD-N terminal (doublet, 29/31 kD), and the VHD (21 kD). Other bands may be alternatively processed forms including heterodimers, trimers and dimers, some of which also exhibit changes associated with decidualisation (black arrows).
(TIF)
