# Correction: Dilated Thin-Walled Blood and Lymphatic Vessels in Human Endometrium: A Potential Role for VEGF-D in Progestin-Induced Break-Through Bleeding

**DOI:** 10.1371/journal.pone.0259337

**Published:** 2021-10-26

**Authors:** Jacqueline F. Donoghue, C. Jay McGavigan, Fiona L. Lederman, Leonie M. Cann, Lulu Fu, Evdokia Dimitriadis, Jane E. Girling, Peter A. W. Rogers

The sixth author’s name is spelled incorrectly. The correct name is: Evdokia Dimitriadis. The correct citation is: Donoghue JF, McGavigan CJ, Lederman FL, Cann LM, Fu L, Dimitriadis E, et al. (2012) Dilated Thin-Walled Blood and Lymphatic Vessels in Human Endometrium: A Potential Role for VEGF-D in Progestin-Induced Break-Through Bleeding. PLOS ONE 7(2): e30916. doi.org/10.1371/journal.pone.0030916

## References

[1] Donoghue JF, McGavigan CJ, Lederman FL, Cann LM, Fu L, Dimitriadis E, et al. (2012) Dilated Thin-Walled Blood and Lymphatic Vessels in Human Endometrium: A Potential Role for VEGF-D in Progestin-Induced Break-Through Bleeding. PLOS ONE 7(2): e30916. 10.1371/journal.pone.0030916 22383980 PMC3284580

