# Supplementary figures and images for: Dilated Thin-Walled Blood and Lymphatic Vessels in Human Endometrium: A Potential Role for VEGF-D in Progestin-Induced Break-Through Bleeding
Source: PLoS One. 2012 Feb 17;7(2):e30916. doi: 10.1371/journal.pone.0030916 (PMC3284580; doi:10.1371/journal.pone.0030916)

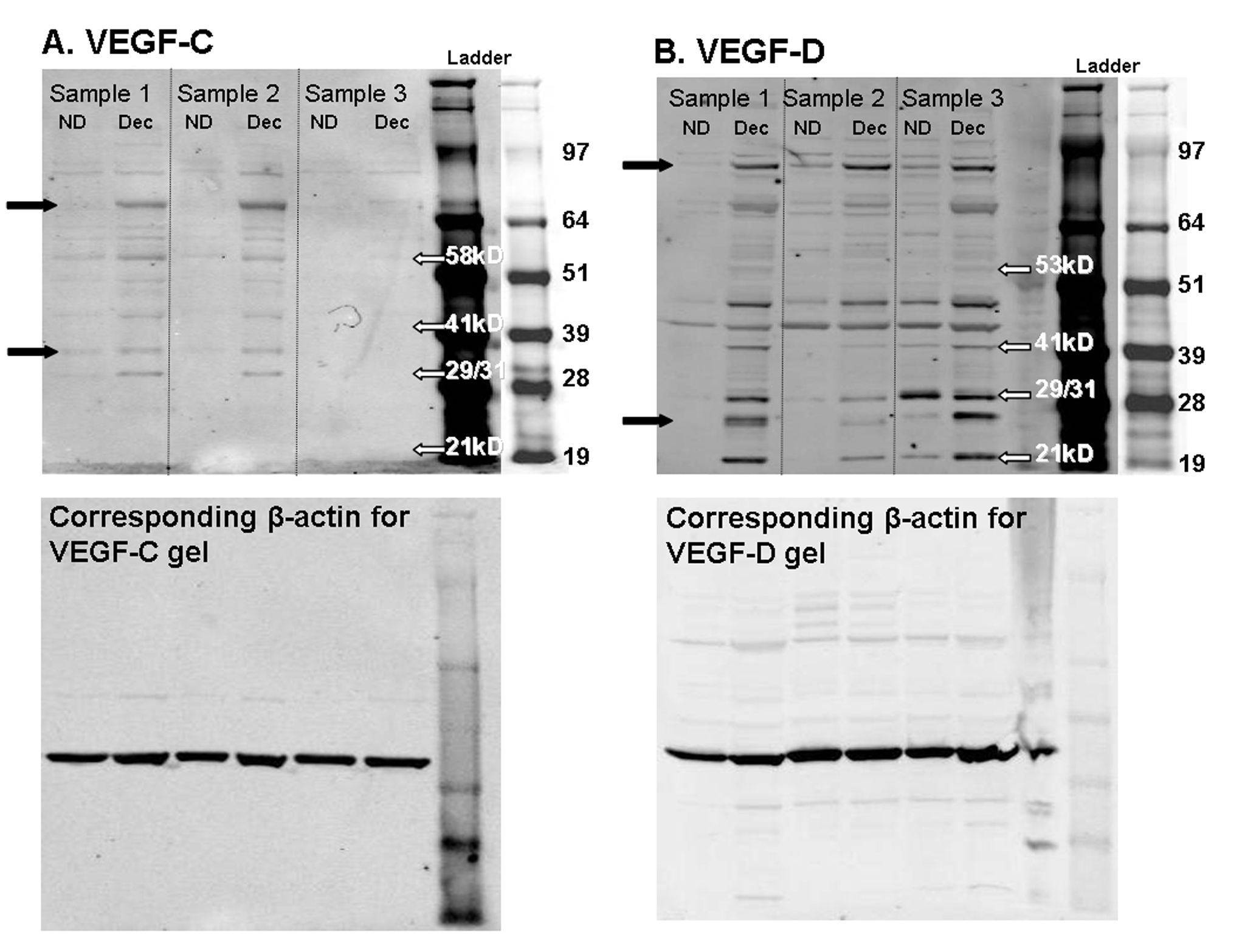

Supplement: Figure S1 — Representative western blots of (A) VEGF-C and (B) VEGF-D protein expression in primary cultures of decidualised (Dec) and non-decidualised (ND) human endometrial stromal cells. White arrows indicate the reported isoforms including the C-VHD-N full length receptor (VEGF-C: 58 kD; VEGF-D: 53 kD; dimers at 105 kD), the VHD-N terminal (doublet, 29/31 kD), and the VHD (21 kD). Other bands may be alternatively processed forms including heterodimers, trimers and dimers, some of which also exhibit changes associated with decidualisation (black arrows). (TIF) [file pone.0030916.s001.tif]
